# On modeling two immune effectors two strain antigen interaction

**DOI:** 10.1186/1753-4631-4-6

**Published:** 2010-11-25

**Authors:** El-Sayed M Ahmed, Hala A El-Saka

**Affiliations:** 1 Mathematics Department, Faculty of sciences, Mansoura 35516, Epypt; 2 Mathematics Department, Damietta Faculty of Science, Mansoura University, 34517, New Damietta, Egypt

## Abstract

In this paper we consider the fractional order model with two immune effectors interacting with two strain antigen. The systems may explain the recurrence of some diseases e.g. tuberculosis (TB). The stability of equilibrium points are studied. Numerical solutions of this model are given. Using integer order system the system oscillates. Using fractional order system the system converges to a stable internal equilibrium. Ulam-Hyers stability of the system has been studied.

## 1 Introduction

Immune system (IS) is known to be multifunctional and multi-pathways i.e. a given function is performed by more than one effector. And each effector, typically perform more than one function [1]. This guarantees to a great extent the resilience of the immune network [2]. Also many antigens evolve with time hence they are multi strains. This explains why some diseases re-appear e.g. tuberculosis (Tb). Therefore modeling the interaction of two immune effectors with two strain antigen is an important problem.

The use of fractional-orders differential and integral operators in mathematical models has become increasingly widespread in recent years [3]. Several forms of fractional differential equations have been proposed in standard models.

Differential equations of fractional order have been the focus of many studies due to their frequent appearance in various applications in fluid mechanics, economic, viscoelasticity, biology, physics and engineering. Recently, a large amount of literatures developed concerning the application of fractional differential equations in nonlinear dynamics [3].

In this paper we study the fractional-order model with two immune effectors interacting with two strain antigen. In sec.2 we present the fractional-order model, study their equilibrium and their local stability and solve it numerically. In sec.3 the Ulam-Hyers stability is presented. In sec. 4 our conclusions are presented.

Now we give the definition of fractional-order integration and fractional-order differentiation:

**Definition 1 **The fractional integral of order *β *∈ *R*^+ ^of the function *f *(*t*)*, t >*0 is defined by

(1) $$I^{\beta }f(t)\;=\int_{0}^{t}\frac{{\left(t-s\right)}^{\beta -1}}{\Gamma (\beta )}f(s)ds$$

and the fractional derivative of order *α *∈ (*n − *1*, n*) of *f *(*t*)*, t >*0 is defined by

(2) $$\begin{array}{l} D_{*}^{\alpha }f(t)\;=\;I^{n-\alpha }D^{n}f(t)\\ D_{*}\;=\;\frac{d}{dt}. \end{array}$$,.

The following properties are some of the main ones of the fractional derivatives and integrals

(see [3-9]).

Let *β, γ *∈ *R*^+ ^and *α *∈ (0, 1). Then

(i) $I_{a}^{\beta }:L^{1}\to L^{1}$ and if *f *(*y*) ∈ *L*^1^, then $I_{a}^{\gamma }I_{a}^{\beta }f(y)=I_{a}^{\gamma +\beta }f(y)$.

(ii) $\lim_{\beta \to n}I_{a}^{\beta }f(y)=I_{a}^{n}f(y)$ uniformly on [a,b], *n *= 1, 2, 3*,...*, where $I_{a}^{1}f(y)=\int_{a}^{y}f(s)ds$.

(iii) $\lim_{\beta \to 0}I_{a}^{\beta }f(y)=f(y)$ weakly.

(iv) If *f*(*y*) is absolutely continuous on [*a, b*], then $\lim_{\alpha \to 1}D_{*}^{\alpha }f(y)=\frac{df(y)}{dy}$.

(v) If *f*(*y*) = *k ≠ 0, k *is a constant, then $D_{*}^{\alpha }k=0$.

The following lemma can be easily proved (see [7]).

**Lemma 1 **Let *β *∈ (0, 1) if *f *∈ *C*[0*, T *], then *I^β^f*(*t*)*|_t _*_= 0 _= 0

### 2 The fractional-order model

Let *x*_1_*, x*_2 _be two strains of an antigen and *y*_1_*, y*_2 _be two immune effectors then the fractionalorder IS model is given by:

(3) $$\begin{array}{l} D_{*}^{\alpha }x_{1}\;=\;a_{1}x_{1}-b_{1}x_{1}y_{1}+c_{1}x_{2}\\ D_{*}^{\alpha }x_{2}\;=\;a_{2}x_{2}-b_{2}x_{2}y_{2}+c_{2}x_{1},\\ D_{*}^{\alpha }y_{1}\;=\;-d_{1}y_{1}+b_{1}x_{1}y_{1},\\ D_{*}^{\alpha }y_{2}\;=\;-d_{2}y_{2}+b_{2}x_{2}y_{2}, \end{array}$$

where 0 *< α ≤ *1 and *a*_1_*, a*_2_*, b*_1_*, b*_2_*, c*_1_*, c*_2_*, d*_1_*, d*_2 _are positive constants. The constants *c*_1_*, c*_2 _are the mutation rates of the antigen strains.

There are several equilibria e.g. the zero equilibrium (0, 0, 0, 0), the interior equilibrium $\left(\frac{d_{1}}{b_{1}},\frac{d_{2}}{b_{2}},\frac{c_{1}d_{2}}{b_{2}d_{1}}+\frac{a_{1}}{b_{1}},\frac{c_{2}d_{1}}{b_{1}d_{2}}+\frac{a_{2}}{b_{2}}\right)\;\text{and }\left(\frac{d_{1}}{b_{1}},-\frac{c_{2}d_{1}}{a_{2}b_{1}},\frac{a_{1}a_{2}-c_{1}c_{2}}{a_{2}b_{1}},0\right),\left(-\frac{c_{1}d_{2}}{a_{1}b_{2}},\frac{d_{2}}{b_{2}},0,\frac{a_{1}a_{2}-c_{1}c_{2}}{a_{1}b_{2}}\right).$

To study the stability we need the Jacobian matrix of (3) given by:

(4) $$\left[\begin{array}{cccc} a_{1}-b_{1}y_{1} & c_{1} & -b_{1}x_{1} & 0\\ c_{2} & a_{2}-b_{2}y_{2} & 0 & -b_{2}x_{2}\\ b_{1}y_{1} & 0 & -d_{1}+b_{1}x_{1} & 0\\ 0 & b_{2}y_{2} & 0 & -d_{2}+b_{2}x_{2} \end{array}\right].$$

It is direct to see that the zero solution is unstable (notice that by definition *a*_1 _*> > c*_1_*, a*_2 _*> > c*_2_*, b*_1 _*> a*_1_*, b*_1 _*> d*_1_*, b*_2 _*> a*_2_*, b*_2 _*> d*_2_).

The numerical simulations of (3) are given in Figures 1,2,3,4,5,6,7,8 for *a*_1 _= 1.0*, a*_2 _= 1.0*, b*_1 _= 2.0*, b*_2 _= 2.0*, c*_1 _= 1.0 *× *10^*−*3^*, c*_2 _= 1.0 *× *10*−*3*, d*_1 _= 1.0*, d*_2 _= 1.0*, x*_1_(0) = 0.3*, x*_2_(0) = 0.5*, y*_1_(0) = 0.5*, y*_2_(0) = 0.3 and different 0 *< α *≤ 1.

**Figure 1:**
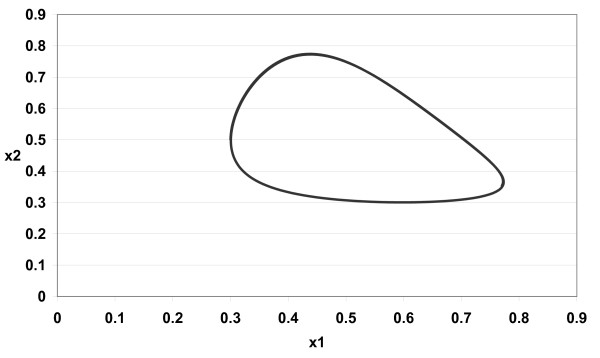
**Figure 1 *α *= 1.0**.

**Figure 2:**
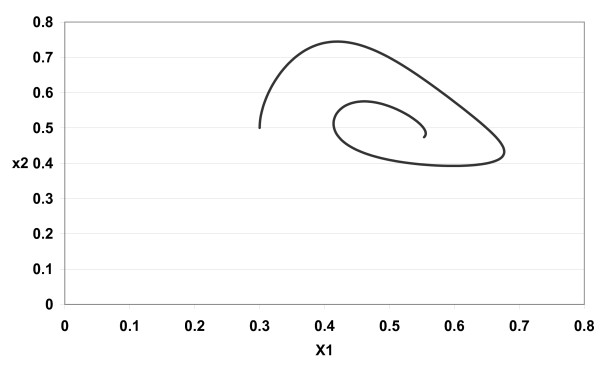
**Figure 2 *α *= 0.9**.

**Figure 3:**
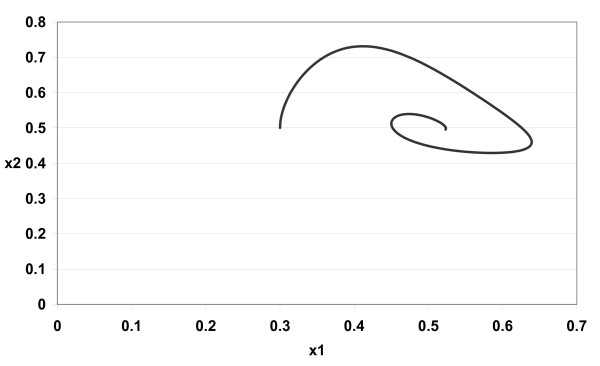
**Figure 3 *α *= 0.85**.

**Figure 4:**
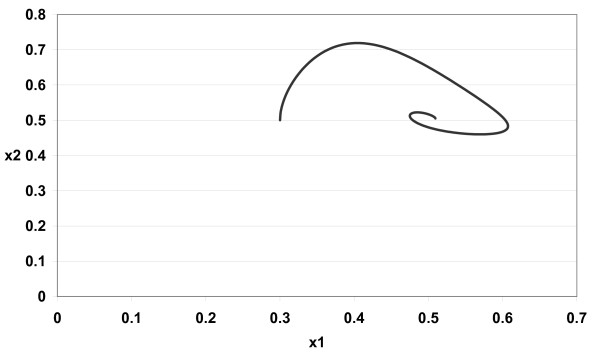
**Figure 4 *α *= 0.8**.

**Figure 5:**
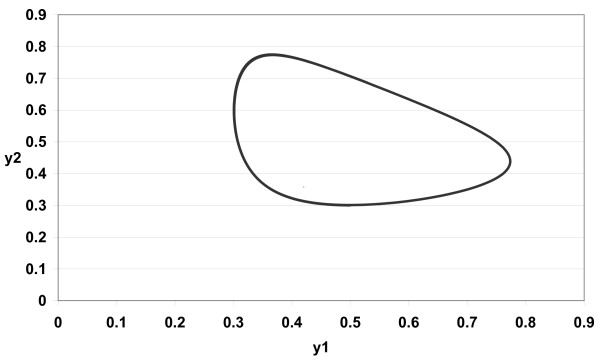
**Figure 5 *α *= 1.0**.

**Figure 6:**
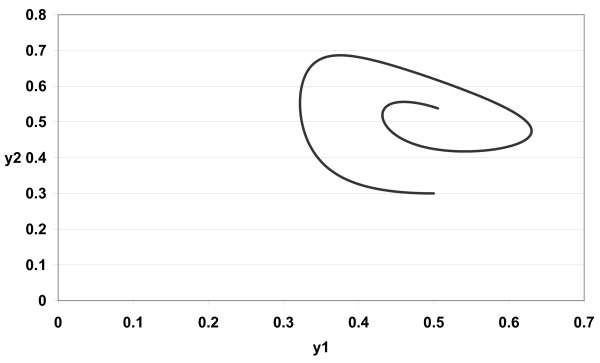
**Figure 6 *α *= 0.9**.

**Figure 7:**
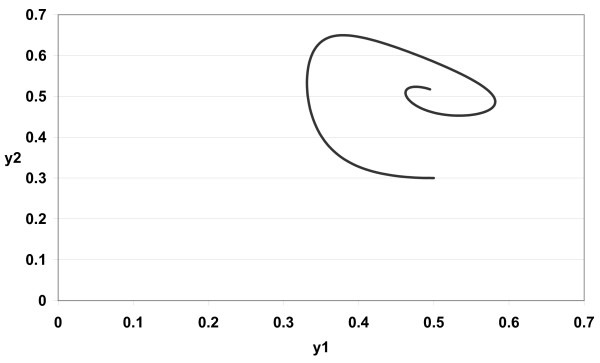
**Figure 7 *α *= 0.85**.

**Figure 8:**
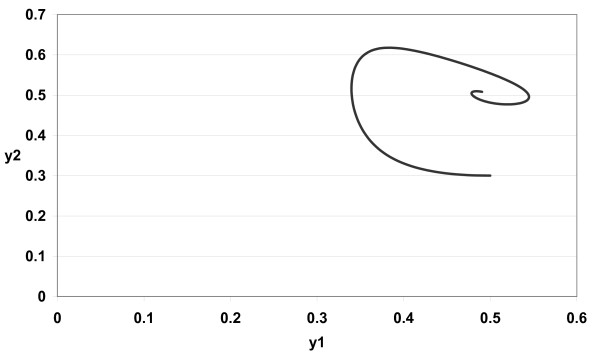
**Figure 8 *α *= 0**.8.

In Figure 1 we take *α *= 1.0. In Figure 2 we take *α *= 0.9. In Figure 3 we take *α *= 0.85. In Figure 4 we take *α *= 0.8. In Figure 5 we take *α *= 1.0. In Figure 6 we take *α *= 0.9. In Figure 7 we take *α *= 0.85. In Figure 8 we take *α *= 0.8. The relation between the two strains of an antigen *x*_1_(*t*) and *x*_2_(*t*) are given in Figures  1,2,3,4 for different 0 *< α *≤ 1. The relation between the two immune effectors *y*_1_(*t*) and *y*_2_(*t*) are given in Figures 5,6,7,8 for different 0 *< α *≤ 1.

The Figures 1,2,3,4,5,6,7,8 show that by using the integer order system (*α *= 1) the system oscillates (Figures 1 and 5) and by using the fractional order system (0 *< α <*1) the system converges to a stable internal equilibrium $\left(\frac{d_{1}}{b_{1}},\frac{d_{2}}{b_{2}},\frac{c_{1}d_{2}}{b_{2}d_{1}}+\frac{a_{1}}{b_{1}},\frac{c_{2}d_{1}}{b_{1}d_{2}}+\frac{a_{2}}{b_{2}}\right)=(0.5,0.5,0.5005,0.5005)$ (Figures 2,3,4, 6,7,8). In Figures 2,3,4 the system converges (0.5, 0.5). In Figures 6,7,8 the system converges to (0.5005, 0.5005).

### 3 Ulam-Hyers stability for systems of equations

Ulam-Hyers stability studies the following question: Suppose one has a function *y*(*t*) which is close to solve an equation. Is there an exact solution *x*(*t*) of the equation which is close to *y*(*t*)?. Mathematically the following system can be studied ([10], [11]):

(5) $$\frac{dx}{dt}=f(x)$$

the system (5) is Ulam-Hyers (UH) stable if it has an exact solution and if ∀*ε >*0 there is

*δ >*0 such that if *x_a_*(*t*) is an approximation for the solution of (5) then there is an exact

solution *x*(*t*) of (5) which is close to *x_a _*i.e.,

(6) $$\begin{array}{l} \left\|\frac{dx_{a}}{dt}-f(x_{a}(t))\right\|\;<\;\delta \Rightarrow \\ \;\;\;\;\;\left\|x(t)\;-x_{a}(t)\right\|<\;\epsilon \forall t>0. \end{array}$$

This definition has applicable significance since it means that if one is studying an UH stable system then one does not have to reach the exact solution (which usually is quite difficult or time consuming). All what is required is to get a function which satisfies (6). UH stability guarantees that there is a close exact solution. This is quite useful in many applications e.g. numerical analysis, optimization, biology and economics etc., where finding the exact solution is quite difficult. It also helps, if the stochastic effects are small, to use deterministic model to approximate a stochastic one.

We begin by realizing that UH stability is independent of the more familiar Lyapunov stability which states that the system (5) is Lyapunov stable if both *x*(*t*)*, y*(*t*) are exact solutions of (5) and for all *ε >*0 there is *δ >*0 such that *| x*(0) *− y*(0) *|< δ *implies *| x*(*t*) *− y*(*t*) *|< ε *for all *t >*0.

A known counter-example proving the independence of the two concepts is the system:

(7) $$\frac{dx}{dt}=ax(t),\;\;\;a>0\text{ constant}$$

whose *x *= 0 solution is Lyapunov unstable while it is UH stable [11].

UH stability has been studied for functional equations [12], and linear differential equations [13].

Now we study local UH stability for nonlinear systems. Consider systems (5), (6), assume

(8) y(t) =x(t) +h(t)

also assume that *h*(*t*) is small hence linearize in it. Substituting in (5), (6) one finally gets

(9) $$h(t)\;\leq \delta f(x)\int \frac{dx}{f^{2}(x)}$$.

Thus we have:

**Proposition (1): **The system (5) is locally UH stable if there is a constant *K *such that

(10) $$\left|f(x)\int \frac{dx}{f^{2}(x)}\right|<K$$.

For a system of equations

(11) $$\frac{dx(i,t)}{dt}=f(i,x_{1},x_{2},\ldots ,x_{n}),\;i=1,2,\;\ldots ,\;n$$

the system is Ulam-Hyers stable if the Jacobian matrix of *f *with respect to *x*_1_*, x*_2_*,..., x_n _*is bounded non-singular. Applying to the model (3) we conclude that the model (3) is Ulam-Hyers stable (with *α *= 1).

## 4 Conclusions

Concluding, the model represents two immune effectors interacting with two strain antigen. The systems may explain the recurrence of some diseases e.g. tuberculosis (TB). Using integer order system the system oscillates. Using fractional order system the system converges to a stable internal equilibrium. Ulam-Hyers stability of the system has been studied.

Now we like to argue that fractional order equations are more suitable than integer order ones in modeling biological, economic and social systems (generally complex adaptive systems) where memory effects are important. From equation (1) it is clear that the fractional order derivative at time *t *depends on the state of the system at all time *t′ ≤ t *hence it naturally accomodates the memory effects. This relation is discussed further in [14].

Also it is known that fractional order derivatives is naturally related to fractals [15]. It is known that fractal structures are abundant in complex adaptive systems.

It is important to notice that Immune system (IS) is known to be multifunctional and multi-pathways i.e. a given function is performed by more than one effector. And each effector, typically perform more than one function [1]. Therefore realistic models require more than one effector for the immune system [16]. This aspect has been included in our model.

## Competing interests

The authors declare that they have no competing interests.

## Authors' contributions

The two authors contributed equally to this article. All authors read and approved the final manuscript.
